# Simultaneous Use of MutS and RecA for Suppression of Nonspecific Amplification during PCR

**DOI:** 10.1155/2013/823730

**Published:** 2013-07-21

**Authors:** Kenji Fukui, Seiki Kuramitsu

**Affiliations:** ^1^RIKEN SPring-8 Center, Harima Institute, 1-1-1 Kouto, Sayo-cho, Sayo-gun, Hyogo 679-5148, Japan; ^2^Science and Technology Entrepreneurship Laboratory, Osaka University, Suita, Osaka 565-0871, Japan; ^3^Department of Biological Sciences, Graduate School of Science, Osaka University, 1-1 Machikaneyama-cho, Toyonaka, Osaka 560-0043, Japan

## Abstract

*Thermus thermophilus* MutS, a thermostable mismatch-recognizing protein, is utilized in PCR to suppress nonspecific amplification by preventing synthesis from mismatched primers. *T. thermophilus* RecA also decreases nonspecific amplification by promoting proper hybridization between the primer and template. We observed that MutS and RecA function under the same reaction conditions and that MutS and RecA do not preclude each other. Furthermore, there were some DNA sequences for which only one of the 2 proteins effectively suppressed nonspecific amplification. The simultaneous use of MutS and RecA is a more attractive error-suppressing technique than the use of either of the 2 proteins alone.

## 1. Introduction

PCR-based technologies are essential for a wide range of biosciences, ranging from basic life sciences to medical diagnoses [1–3]. The efficiency and reliability of those technologies are often decreased by nonspecific amplifications resulting from mishybridization of primers. Recently, we developed a technique to suppress nonspecific amplification by adding a thermostable mismatch-recognizing protein *Thermus thermophilus* MutS (ttMutS) in PCR mixture [4]. MutS is a DNA mismatch repair protein, which recognizes a variety of DNA mismatches such as base-base mismatches and base insertion loops [5, 6]. MutS is thought to bind tightly to the mismatched bases that are generated by mishybridization between primer and template to block the association of a DNA polymerase. Before we established the MutS-based error-suppressing technique, it had been reported that *T. thermophilus* RecA (ttRecA) protein can be used to decrease nonspecific amplification during PCR, which drastically enhances the accuracy of multiplex PCR [7]. RecA is known to be a key enzyme in homologous recombination of DNA molecules, which catalyzes pairing of 2 homologous sequences [8]. The ttRecA protein enhances the proper pairing between the primer and template to suppress nonspecific amplification. Thus, the mechanisms by which ttMutS and ttRecA suppress nonspecific amplification are quite different. In this study, we investigated the difference in the effects and properties of ttMutS and ttRecA in order to evaluate the possibility of simultaneous use of both proteins.

## 2. Materials and Methods

### 2.1. Overexpression and Purification of Proteins

*Thermus thermophilus *MutS (ttMutS) was prepared as previously described [4]. A DNA fragment-expressing *T. thermophilus* RecA (ttRecA) was generated by PCR using the *T. thermophilus *HB8 genomic DNA as a template. The following primer pairs were used for amplification of the fragment: 5′-ATATCATATGGACGAGAGCAAGCGCAAGGC-3′ and 5′-ATATGGATCCTTATTACTCCCCCTCGTCCTCGCCC-3′. The forward and reverse primers contained NdeI and BamHI restriction sites, respectively (underlined). The amplified fragment was ligated into the NdeI and BamHI sites of pET-11a (Novagen, Madison, WI, USA) to obtain pET-11a/*T. thermophilus recA*. Sequence analysis revealed that the construction was error-free. *E. coli *BL21 (DE3) was transformed with pET-11a/*T. thermophilus recA* and then grown at 37°C in 1.5 liters of YT medium containing 50 *μ*g/mL of ampicillin. When the density of cultures reached 2.9 × 10^8^ cells/mL, isopropyl *β*-d-thiogalactopyranoside was added to 100 *μ*M. The cells were grown at 37°C for 3 h after induction and harvested by centrifugation. The cells were lysed by sonication in 50 mM Tris-HCl (pH 7.5) containing 100 mM NaCl and 1.5 M KCl and then heated to 60°C for 60 min. After centrifugation at 48,000 ×g for 20 min, the supernatant was isolated, and ammonium sulfate was added to a final concentration of 1.5 M. The solution was loaded onto a TOYOPEARL-Phenyl column (12 mL, TOSOH, Tokyo, Japan) preequilibrated with 50 mM Tris-HCl (pH 8.0) containing 1.5 M ammonium sulfate. The column was washed with the same buffer and then eluted with 1.5–0 M ammonium sulfate gradient in the same buffer. The fraction containing ttRecA was detected by SDS-PAGE and dialyzed against 50 mM Tris-HCl (pH 8.0). The solution was loaded onto a TOYOPEARL-DEAE column (12 mL, TOSOH) preequilibrated with 50 mM Tris-HCl (pH 8.0). The column was washed with the same buffer and then eluted with 0–1.5 M KCl gradient in 50 mM Tris-HCl (pH 8.0). 

### 2.2. PCR Using ttRecA and ttMutS Proteins

The 5′-terminal 423-bp region of the *ttha1806* gene was amplified using the primers 5′-GAGACCACCCGTAGGCGGCT-3′ and 5′-CTTAAGGGGCCTCGCGCTCT-3′; a 1,278-bp region of the *ttha1548* gene was amplified using 5′-GAGGAGGTGCTCTACGTGGGCAAGGCC-3′ and 5′-GGGAAGGTCCTTGAGGCTTCCCGTGTAGC-3′; a 611-bp region of the *ttha0122* gene was amplified using 5′-ATGTTCCTGAGGATAGACCGCC-3′ and 5′-ATCTCCACCCCGGTGAGGC-3′; and a 466-bp region of the *ttha1300* gene was amplified using 5′-ATGCCCGCCATGGAAGTGG-3′ and 5′-TGAGCGCCTTCAGGGCCT-3′. The primers were synthesized by BEX Co. (Tokyo, Japan). The reactions were performed in 1 × Takara GC I buffer (Takara, Shiga, Japan) containing 0.06 units/*μ*L Takara LA Taq (Takara), 5 ng/*μ*L* T. thermophilus *HB8 genomic DNA, 400 nM primers, 100 *μ*M CoCl_2_, and 400 *μ*M dATP, dTTP, dCTP, and dGTP (Takara) in the presence of various concentrations of ttRecA and ttMutS. Thirty PCR cycles were run using ASTEC OC707 (ASTEC, Tokyo, Japan): denaturation step at 95°C for 1 min, annealing at 58°C for 1 min, and extension at 70°C for 2 min for amplification of the *ttha1806 *gene; and denaturation step at 95°C for 1 min, annealing at 50°C for 1 min, and extension at 70°C for 2 min for amplification of the *ttha0122*, *ttha1300*, and *ttha1548* genes. A 10 *μ*L reaction solution was mixed with 1 *μ*L of loading buffer (50% [v/v] glycerol, 0.9% [w/v] SDS, and 0.05% [w/v] bromophenol blue) and electrophoresed on a 1.5% agarose gel in 1 × TBE buffer. The gel was stained with ethidium bromide, and the products were visualized under ultraviolet light at 254 nm. The relative amounts of the amplified DNA products were quantified using the ImageJ software [10].

## 3. Results and Discussion

**Figure 1:**
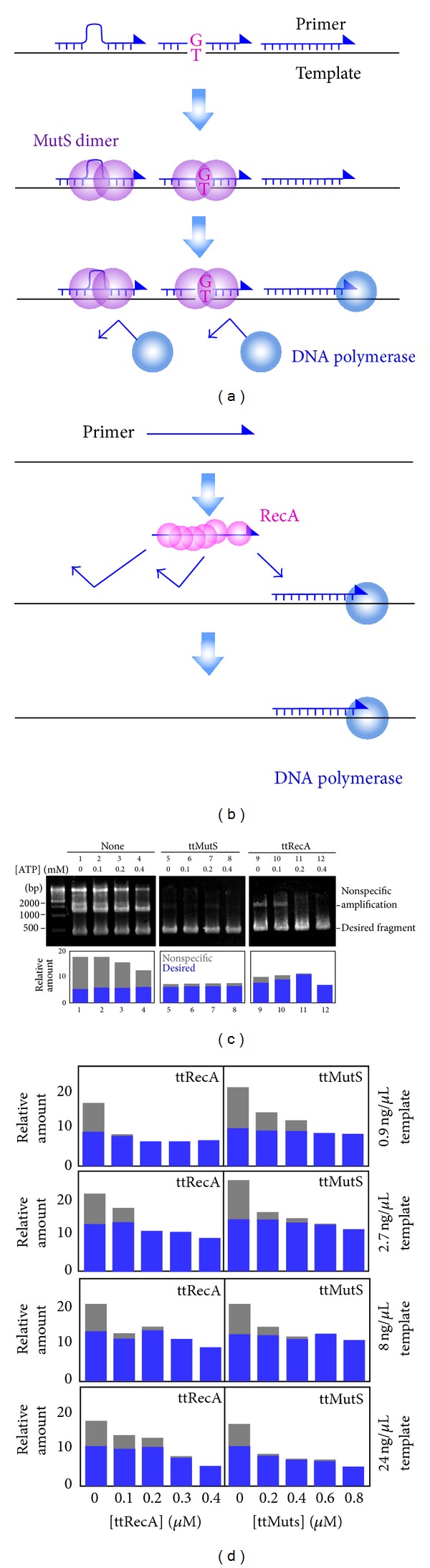
The error-suppressing effects of ttRecA and ttMutS in the presence of ATP. (a) A schematic representation for the mechanism by which ttMutS suppresses nonspecific amplifications during PCR. A ttMutS dimer recognizes mismatched bases generated by mishybridization of the primer and blocks the approach of DNA polymerase. (b) A schematic representation for the mechanism by which ttRecA suppresses nonspecific amplification during PCR. ttRecA promotes proper priming for PCR. (c) A 423 bp region of the *ttha1806* gene was amplified by using Takara LA Taq in the presence of 0 to 0.4 mM ATP. Lanes 1–4, 5–8, and 9–12 are the results of the reaction without ttMutS or ttRecA, with 0.8 *μ*M ttMutS, and with 0.4 *μ*M ttRecA, respectively. The amounts of the amplified fragments were quantified by using the ImageJ software [9] and are shown as bar graphs in the lower panels, where gray and blue indicate nonspecific and desired amplifications, respectively. (d) A 423 bp region of the *ttha1806* gene was amplified by using Takara LA Taq in the presence of 0.9, 2.7, 8.0, or 24 ng/mL template DNA (*T. thermophilus* HB8 genomic DNA). The relative amounts of the amplified fragments are shown. Gray and blue bars indicate nonspecific and desired amplifications, respectively.

As has been previously described, RecA catalyzes the homologous pairing of DNA molecules in an ATP-dependent manner [11–13], and ttRecA requires ATP for the error-suppressing effect during PCR [7]. On the other hand, ATP is not essential for the error-suppressing effect of ttMutS during PCR [4]. However, it is known that MutS has a Walker's A-type ATPase motif [14, 15] and that the conformation and function of MutS are greatly affected by the binding and hydrolysis of ATP. Binding of ATP triggers the formation of sliding-clamp mode of MutS, and enhances the dissociation of MutS from a mismatch [10, 16]. Therefore, we examined whether or not ATP interferes with the error-suppressing effect of MutS during PCR. The 423-base pair (bp) region of the *ttha1806* gene was amplified by Takara LA Taq using *T. thermophilus* HB8 genomic DNA as the template. Without ttMutS and ttRecA, 1000- to 5000-bp DNA fragments were amplified nonspecifically (Figure 1(c)). ttMutS showed a significant suppressing effect on the nonspecific amplifications in the presence of 0 to 0.4 mM ATP (Figure 1(c)). The previous reports described that 0.4 mM ATP is enough to ensure the maximum effect of ttRecA [7], which was also supported by our results (Figure 1(c)). It also should be noted that 0.4 mM ATP did not influence the specificity of PCR amplification (Figure 1(c), left panel). Thus, ttMutS and ttRecA function under the same reaction conditions. We also demonstrated that 0.4 *μ*M ttRecA and 0.8 *μ*M ttMutS were suitable for suppressing the nonspecific amplifications during PCR, with a wide range of template concentrations (0.9 to 24 ng/*μ*L) (Figure 1(d)). Since ttMutS and ttRecA are not stable at the temperature over 90°C, the error-suppressing effect may be improved by supplementing the reaction with additional proteins at the middle of the PCR cycle. However, as shown in our data, we were able to obtain satisfactory effect without the supplementation when we use 0.4 *μ*M ttRecA and 0.8 *μ*M ttMutS. The supplementation of the additional proteins may be required when we cannot obtain enough effect under these conditions. 

**Figure 2:**
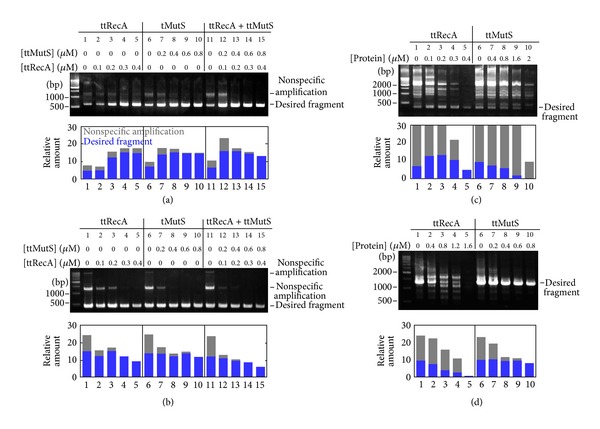
Various target sequences were amplified in the presence of ttRecA and ttMutS. (a) A 611 bp region of the *ttha0122 *gene was amplified from* T. thermophilus *HB8 genomic DNA template in the presence of ttRecA and ttMutS. The amounts of amplified fragments were quantified and are shown as bar graphs in the lower panel. Gray and blue bars indicate nonspecific and desired amplifications, respectively. (b) A 423 bp region of the *ttha1806 *gene was amplified from* T. thermophilus *HB8 genomic DNA template in the presence of ttRecA and ttMutS. (c) A 466 bp region of the *ttha1300* gene was amplified from* T. thermophilus *HB8 genomic DNA in the presence of ttRecA or ttMutS. Note that relatively high concentrations of ttMutS were used here. (d) A 1,278 bp region of the *ttha1548* gene was amplified in the presence of ttRecA or ttMutS. Note that relatively high concentrations of ttRecA were used here.

To determine whether ttRecA and ttMutS preclude the effect of each other, a 611-bp region of the *ttha0122* gene was amplified by Takara LA Taq using *T. thermophilus* HB8 genomic DNA as a template. In the absence of ttRecA and ttMutS, relatively weak amplification of the target sequence and the nonspecific amplification of 1000- to 1500-bp were observed (Figure 2(a)). Both ttRecA and ttMutS enhanced the amplification of the desired fragment and suppressed the nonspecific amplification (Figure 2(a)). The error-suppressing effect was retained when both ttRecA and ttMutS were present (Figure 2(a)). The same result was also obtained when the 423-bp region of the *ttha1806* gene was amplified in the presence of ttRecA and aqMutS (Figure 2(b)). These findings suggest that ttRecA and ttMutS can be used simultaneously without any interfering effects.

In order to evaluate the validity of the simultaneous use of ttRecA and ttMutS, we investigated the difference in the specificities of ttRecA and ttMutS towards the sequences to be amplified. If their error-suppressing effects show different specificities for the target sequences, simultaneous use would cover a wider range of sequences than the single use of ttRecA or ttMutS would. As shown in Figure 2(c), nonspecific amplification upon the amplification of a 466-bp region of the *ttha1300* gene was effectively suppressed by ttRecA but not by ttMutS. In contrast, ttMutS showed a strong error-suppressing effect on the amplification of a 1,278-bp region of the *ttha1548* gene, whereas ttRecA did not (Figure 2(d)). These results clearly indicate that ttRecA and ttMutS have distinct specificities against the target sequences. This is expected because ttRecA and ttMutS suppress nonspecific amplification in different ways. 

## 4. Conclusions

In this study, we clarified that ttMutS and ttRecA do not preclude each other and there are some sequences for which only one of the 2 proteins efficiently suppresses nonspecific amplification. Our results in this study suggest that simultaneous use of ttMutS and ttRecA is better than the use of either ttMutS or ttRecA alone.
